# Antineutrophil Cytoplasmic Antibody (ANCA)-Associated Renal Vasculitis Following COVID-19 Vaccination: A Case Report and Literature Review

**DOI:** 10.7759/cureus.30206

**Published:** 2022-10-11

**Authors:** Khalid Uddin, Khalid H Mohamed, Adesola A Agboola, Warda A Naqvi, Helai Hussaini, Alaa S Mohamed, Muhammad Haseeb, Hira Nasir

**Affiliations:** 1 Neurology, Henry Ford Health System, Detroit, USA; 2 Neurology, Sheffield Teaching Hospitals NHS Foundation Trust, Sheffield, GBR; 3 Pathology and Laboratory Medicine, Dele Hospitals, Lagos, NGA; 4 Infectious Diseases, Shifa International Hospital, Islamabad, PAK; 5 Internal Medicine, Toronto General Hospital, Toronto, CAN; 6 Neurology, Augusta University, Augusta, USA; 7 Internal Medicine, Jinnah Hospital Lahore, Lahore, PAK; 8 Internal Medicine, Mayo Hospital, Lahore, PAK

**Keywords:** covid-19, vasculitis, renal vasculitis, antineutrophil cytoplasmic antibody, anca-associated vasculitis, covid-19 vaccine, covid-19 vaccine complication, pauci-immune anti-neutrophil cytoplasmic antibody (anca)-associated vasculitis (aav), pauci-immune glomerulonephritis (gn)

## Abstract

Antineutrophil cytoplasmic antibody (ANCA)-associated vasculitis (AAV) is an immune-mediated disorder of small and medium-sized vessels, characterized by the production of autoantibodies that target the neutrophilic antigens leading to mononuclear cell infiltration and destruction of blood vessels in lungs, skin, and kidneys. Although rare, the coronavirus disease 2019 (COVID-19) vaccine may trigger autoimmune vasculitis. We report a rare case of ANCA-associated renal vasculitis following COVID-19 vaccination in a 59-year-old male who presented with flu-like symptoms and deranged renal function tests. He received his second dose of the Pfizer COVID-19 vaccine 17 days ago. His clinical picture, serological testing, and radiological imaging were concerned with glomerular disease. His serum was positive for ANCAs, and the renal biopsy specimen revealed pauci-immune glomerulonephritis. He was diagnosed with AAV-associated renal vasculitis following COVID-19 vaccination because no other etiology was identified. His clinical improvement after starting rituximab and steroids reinforced the diagnosis.

## Introduction

Antineutrophil cytoplasmic antibody (ANCA)-associated vasculitis (AAV), an autoimmune disease, represents a group of small vessel vasculitis hallmarked by autoantibodies that target neutrophilic antigens, mainly leukocyte proteinase 3 (PR3) and myeloperoxidase (MPO) [1]. AAV is an immune-mediated disorder characterized by granulomatous and neutrophilic tissue inflammation and small vessel injury in any body organ system, predominantly in the lungs, kidneys, and skin [2]. ANCA-associated renal vasculitis may manifest with proteinuria, hematuria, deranged renal function tests, and constitutional signs and symptoms. It is believed that several factors, including vaccines, infectious agents, specific medications, and environmental exposure, may induce AAV and autoimmunity [1,2]. Vaccine-induced AAV has also been documented in the literature [3]. ANCA-associated renal vasculitis caused by the coronavirus disease 2019 (COVID-19) vaccine is rarely reported in the literature [4]. We report a case of AAV-associated renal vasculitis following COVID-19 vaccination.

## Case presentation

A 59-year-old male with a past medical history of hypertension and ischemic heart disease presented with fever, malaise, and polyarthralgia for the last five days. His fever was mild and intermittent, associated with nausea and anorexia. He received his second dose of the Pfizer COVID-19 vaccine 17 days ago. He was compliant with his medications. He had no history of trauma, travel, alcohol abuse, and substance abuse. He had no history of smoking and COVID-19 infection. He also had no family of autoimmune disease.

On examination, he was febrile (99°F) with a heart rate of 89/minute, blood pressure of 130/85 mmHg, and respiratory rate of 21/minute. His physical examination was unremarkable for synovitis involving multiple joints except for mild pain on active movements. On auscultation, he had normal vesicular breathing and regular heart sounds. The initial laboratory studies revealed low hemoglobin levels and deranged renal function tests (Table 1). His chest X-ray was unremarkable. Urine analysis showed proteinuria, positive occult blood, red cell casts, and microscopic hematuria. His spot urinary protein to creatinine ratio was 0.90 mg/g. He was managed conservatively with intravenous hydration. His blood and urine cultures were negative. An abdominal ultrasound was performed, which showed parenchymal echogenicity and normal renal dimensions.

**Table 1:** Results of initial laboratory tests.

Parameter	Lab result (reference range)
Hemoglobin	9.9 g/dl (13.2-16.6)
Platelet count	201,000 cells/mm^3^ (150,000-350,000)
White blood cell count	8900 cells/mm^3^ (4000-11000)
Red blood cell count	4.31 million cells/ul (4.20-5.65)
Blood urea nitrogen	59 mg/dl (13-21)
Serum creatinine	3.5 mg/dl (0.7-1.2)
Brain natriuretic peptide	2100 pg/ml (<450)
Erythrocyte sedimentation rate	25 mm/hr (0-22)

His serum creatinine remained elevated despite intravenous hydration, and he underwent serological testing, which showed positive ANCA titers (>131 IU/ml). His serology titer for perinuclear ANCA (p-ANCA; anti-PR3 antibody) was 69 IU/ml and for cytoplasmic ANCA (c-ANCA; anti-MPO antibody) was 1:120. He was negative for other antibodies, and immunoglobulins levels and blood complement C3 and C4 were within the normal range (Table 2).

**Table 2:** Results of serological testing. GBM: glomerular basement membrane; dsDNA: double-stranded deoxyribonucleic acid; Ig: immunoglobulin; ANA: antinuclear antibodies.

Parameter	Lab result
Anti-GBM antibody	Negative
ANA	Negative
Anti-dsDNA antibody	Negative
C3 factor	114 mg/dl (80-178)
C4 factor	21 mg/dl (12-42)
IgA	1.9 g/L (0.8-3.0)
IgG	7.1 g/L (6-16)
IgM	101 mg/dl (40-240)

**Figure 1:**
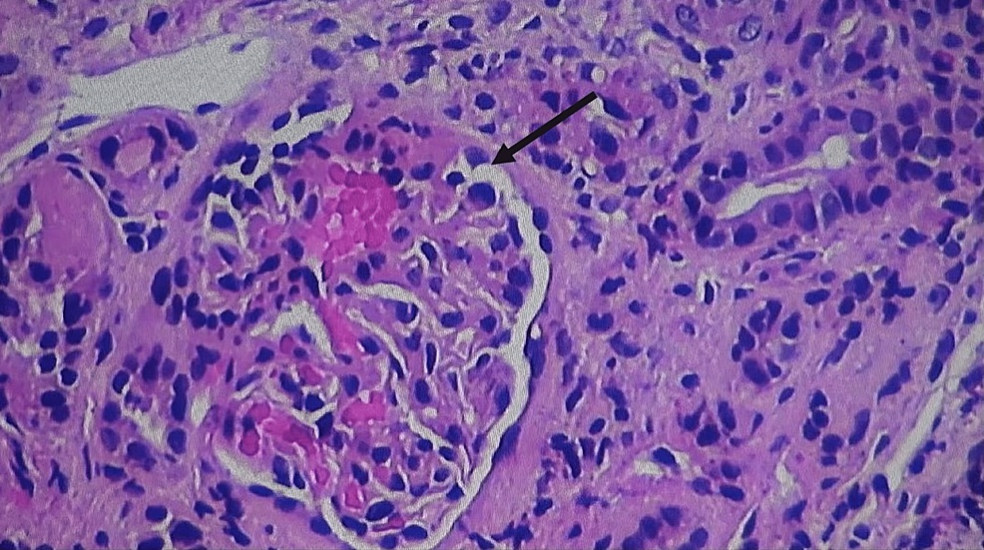
Renal biopsy demonstrating diffuse glomerular sclerosis, fibrin deposits, neutrophilic infiltration, and tubal interstitial scarring with casts in the lumina.

A renal biopsy was performed, which showed diffuse global sclerosis, fibro-cellular crescents in glomeruli, arterial hyalinosis, moderate arteriosclerosis, and focal tubal interstitial scarring (Figure 1). Based on serology and renal biopsy findings, he was diagnosed with pauci-immune glomerulonephritis (GN) due to COVID-19 vaccination because no other etiology was identified. He was managed with pulse therapy of methylprednisolone 1 g daily for three days and intravenous rituximab 375 mg/m^2^, followed by a tapering dose of prednisone 1 mg/kg daily. A follow-up dose of rituximab was given two weeks apart. His serum creatinine and proteinuria improved on his recent follow-up, and his clinical symptoms gradually improved (Table 3).

**Table 3:** Clinical laboratory findings after vaccination. WBCs: white blood cells; RBCs: red blood cells; Cr: creatinine; HPF: high-power field.

Days after vaccination		+17	+26	+36	Reference
Serum Cr, mg/dl		3.25	3.00	2.75	0.5-0.9
Serum sodium, mEq/L		135	132	141	135-145
Serum potassium, mEq/L		4.1	4.6	3.9	3.5-5.0
Serum chloride, mEq/L		101	98	99	98-107
Urinalysis					
	Specific gravity	1.015	1.016		1.010-1.030
	Protein	2+	1+	-	Negative
	Glucose	Trace	Trace	-	Negative
	Occult blood	2+	1+	-	Negative
	Nitrite	Negative	Positive	-	Negative
	RBCs/HPF	0-29	5-9	-	0-5
	WBCs/HPF	Numerous	Many	-	0-6

## Discussion

AAV most commonly affects the kidneys, and the disease outcomes are mainly affected by the degree of renal impairment at diagnosis and treatment response, and in older age, renal failure is primarily caused by AAV in the absence of any obvious trigger [5]. ANCA can be limited to the renal system alone or may affect the skin, brain, and lungs in various combinations. Manifestations of renal disease include flu-like syndromes such as malaise, fever, myalgia, and arthralgia. Laboratory investigations typically revealed elevated serum creatinine, moderate proteinuria, hematuria, and red blood cell casts [2,5]. The etiology and pathogenesis of AAV are multifactorial, with contributions from infection, drugs, genetic factors, environmental exposure, and vaccines [1]. Although rare, vaccine-induced AAV has been highlighted in the literature. Jeffs et al. reported a case of AAV induced by the influenza vaccine [3]. The clinical cases of AAV induced by the COVID-19 vaccine have also been published. We have tabulated the published cases of ANCA-associated renal vasculitis following COVID-19 vaccination in Table 4.

**Table 4:** Reported cases of renal-associated AAV induced by COVID-19 vaccination. ANCA: antineutrophil cytoplasmic antibody; AAV: ANCA-associated vasculitis; MPO: myeloperoxidase; PR3: proteinase 3.

Authors	Age/sex	COVID-19 vaccine type	Onset of symptoms	Clinical presentation	ANCA positivity	Management
Shakoor et al. [4]	79/F	Pfizer-BioNTech (second dose)	2 weeks	Weakness, upper thigh pain, acute kidney injury	MPO-ANCA	Steroids, cyclophosphamide
Dube et al. [6]	29/F	Pfizer-BioNTech (second dose)	16 days	Acute kidney injury	MPO-ANCA	Steroids, rituximab, cyclophosphamide
Takenaka et al. [7]	75/F	Pfizer-BioNTech (first dose)	4 days	Blurred vision	MPO-ANCA	Steroids
Sekar et al. [8]	52/M	Moderna (second dose)	2 weeks	Headache, weakness, acute kidney injury	PR3-ANCA	Steroids, cyclophosphamide
Anderegg et al. [9]	81/M	Moderna (second dose)	Not reported	Flu-like symptoms, acute kidney injury	PR3-ANCA	Steroids, cyclophosphamide, plasma exchange therapy
Al-Yafeai et al. [10]	62/F	Pfizer-BioNTech (first dose)	4 weeks	Weakness, hematemesis, arthralgia, acute kidney injury	PR3-ANCA	Steroids, cyclophosphamide, plasma exchange therapy

The molecular mechanisms of vaccine-induced AAV are not well defined. It is believed that adjuvant molecules of the vaccine may trigger autoimmunity [8]. Other mechanisms may involve viral persistence followed by epitope spreading, and bystander killing may induce an immune-mediated response leading to AAV [11,12]. Molecular mimicry, defective neutrophilic apoptosis, complement system activation, systemic inflammatory response, and polyclonal activation of immune cells triggered by neutrophil extracellular trap formation and pro-inflammatory protein may also induce vasculitis in genetically susceptible individuals [13]. A vaccine may induce differential stimulation of dendritic and myeloid cells, activating a cascade of pathways to produce autoinflammation [8,9,12].

Our patient was diagnosed with pauci-immune GN following the COVID-19 vaccine, and his improvement after starting steroids and rituximab provides a temporal association between renal AAV and COVID-19 vaccination. Despite a limited number of cases reported, there is a temporal correlation between vasculitis and vaccination; whether it equals causation is debatable. COVID-19 vaccine-induced AAV is uncommon, and mass vaccination against COVID-19 infection worldwide offers the proper ground to observe and understand the correlation of vasculitis with the vaccine.

## Conclusions

Despite the substantial benefits of the COVID-19 vaccine, autoimmune processes, particularly AAV, have been highlighted post-vaccination. AAV is a rare and life-threatening complication, and the objective of our case is to inform the clinicians to encourage the hasty recognition and diagnosis of vasculitis in timely correlation with the COVID-19 vaccine, a complete workup including imaging modalities of possible concurrent triggers, as well as timely appropriate management once found.
